# Decrypting the Molecular Mechanistic Pathways Delineating the Chemotherapeutic Potential of Ruthenium-Phloretin Complex in Colon Carcinoma Correlated with the Oxidative Status and Increased Apoptotic Events

**DOI:** 10.1155/2020/7690845

**Published:** 2020-05-31

**Authors:** Guoguo Jin, Zhenjiang Zhao, Tania Chakraborty, Aikyadeep Mandal, Arka Roy, Souvik Roy, Zhiping Guo

**Affiliations:** ^1^Laboratory of Bone Tumor, The Henan Luoyang Orthopedic Hospital, Zhengzhou, Henan 450000, China; ^2^Department of Radiology, The Henan Luoyang Orthopedic Hospital, Zhengzhou, Henan 450000, China; ^3^Department of Pharmacy, NSHM Knowledge Campus-Kolkata, 124 B.L., Saha Road, Kolkata, 700053 West Bengal, India; ^4^Department of Orthopeadic Surgery, The Henan Luoyang Orthopedic Hospital, Zhengzhou, Henan 450000, China

## Abstract

To explore fresh strategies in colorectal cancer (CRC) chemotherapy, we evaluated the capability of the ruthenium-phloretin complex in exterminating colon cancer by effectively addressing multiple apoptotic mechanisms on HT-29 cancer cells together with an animal model of colorectal cancer activated by 1,2-dimethylhydrazine and dextran sulfate sodium. Our current approach offers tangible evidence of the application of the ruthenium-phloretin complex in future chemotherapy. The complex triggers intrinsic apoptosis triggered by p53 and modulates the Akt/mTOR pathway along with other inflammatory biomarkers. The ruthenium-phloretin complex has been synthesized and successfully characterized by numerous spectroscopic methodologies accompanied by DPPH, FRAP, and ABTS assays assessing its antioxidant potential. Studies conducted in human cell lines revealed that the complex improved levels of p53 and caspase-3 while diminishing the activities of VEGF and mTOR, triggers apoptosis, and induces fragmentation of DNA in the HT-29 cells. Toxicity studies were conducted to identify the therapeutic doses of the novel complex in animal models. The outcomes of the in vivo report suggest that the complex was beneficial in repressing multiplicity of aberrant crypt foci as well as hyperplastic lesions and also promoted increased levels of CAT, SOD, and glutathione. In addition, the ruthenium-phloretin complex was able to control cell proliferation and boosted apoptotic outbursts in cancer cells associated with the increase in cellular response towards Bax while diminishing responses towards Bcl-2, NF-*κ*B, and MMP-9. Our observations from the experiments deliver testament that the ruthenium-phloretin complex has the potential to act as a promising chemotherapeutic agent in colorectal cancer because it can affect the growth of ACF and hyperplastic abrasions in the colon tissues by evoking cell death.

## 1. Introduction

Colorectal cancer (CRC) is the second most common cancer in the world, accounting for 8.5% of all deaths due to cancer [1]. Nations such as the Republic of Korea, Singapore, the Philippines, Thailand, China, and India have seen a steady increase in colorectal cancer prevalence in the recent past. The sharp increase in colorectal cancer incidences across the developing countries of pan-Asia could have been due to the spurt of economic development including a transformation of ecology and lifestyle [2]. Throughout China, the rise in the annual rate of colon cancer and mortality has gone from 14.25 per 100,000 in 1990 to 25.27 per 100,000 in 2016 [3]. The annual colon cancer incidence rates (AARs) in India in 2017 is about 4.4 per 100,000 males and 3.9 per 100,000 females [4]. Contrary to the lower prevalence of CRC, the 5-year rate of survival remains extremely poor, shockingly also for localized cases, creating the impression of a substantial shortage in adequate intervention and diagnosis [5]. Although there are several alternatives available for treating this disease, such as surgery, chemotherapy, radiation therapy, and immunotherapy, recovery rates are not all that encouraging [6]. Chemotherapy still remains to be the predominant therapeutic alternative for CRC, and successful approaches substantially prolong the longevity and ensure well-being of patients in CRC [7]. However, frightening adverse effects and drug resistance limit the implementation of extended chemotherapy in CRC.

Around 1978, cisplatin (a platinum derivative) led everyone to a revolutionary era of cancer therapy, culminating in the application of platinum-based chemotherapy for a considerable patient population [8]. Nevertheless, typical repercussions of platinum-based chemotherapy include nausea, vomiting, gastrointestinal discomfort, and reduced blood levels. Multidrug resistance (MDR) is another crucial aspect for the failure of platinum-based chemotherapy resulting from cross-resistance to many other nonrelated agents structurally, chemically, and functionally [9]. These dramatic times call for an effective drug with enhanced cellular concentration at safe doses for the complete eradication of carcinogenic cells. Several other metal-based drugs often do not deteriorate rapidly in vivo as compared to platinum drugs, which are absolutely essential for the transmission of the drugs to effective regions in order to combat the disease and fight toxicity [10]. Lately, ruthenium (Ru) has stimulated our expectations, in metal-based therapies, owing to its assorted oxidative stages (II, III, and IV); improved consistency, specificity, oxidation, and reduction potential; DNA intercalation; ligand exchange kinetics; and iron mimicking behavior [11]. Studies on derivatives centered around ruthenium have demonstrated very beneficial results in demonstrating the anticancer potential of the metal [12–15]. NAMI-A is one of the forerunners of phase II chemotherapy trials focused on ruthenium (III)-based drugs [16]. Additional compounds belonging to this category KP1019 [17] and NKP1339 (a sodium salt of KP1019) are also accredited for clinical use [18]. Interestingly certain studies point out that metal-drug complexes are better suited to alternative chemotherapy than pure metals [19, 20].

The colorectal cancer-diet correlation is a well-documented one that has been established over the last 25 years [21]. Various population-based research denotes that higher fiber-rich foods/fruit consumption along with lower amounts of red meat, fat, and alcohol ingestion is all linked to lower incidences of CRC [22, 23]. Apple is one such dietary source offering a plethora of antioxidants and anti-inflammatory agents. Among several polyphenols present in cross-resistance is phloretin [2′,4′,6′-trihydroxy-3-(4-hydroxyphenyl)-propiophenone], which has gained substantial recognition as an antineoplastic agent [24]. Tanaka et al.'s research spearheaded the demonstration that phloretin was capable of preventing the neoplastic progression of Balb/3T3 cells when subjected to a model tumor-inducing agent, 12-O-tetradecanoyl phorbol-13 acetate (TPA) [25]. Ensuing findings have confirmed that phloretin possesses activities triggering antioxidation, anti-inflammation, antiproliferation, and apoptosis in vivo.

Additionally, phloretin was also able to prevent the progression of xenograft tumors in nude mice when used independently or in conjunction with standard chemotherapy [26–29].

One of the most elementary detectable lesions in animal CRC prototypes is the aberrant crypt foci or ACF that is associated with the colon cancer progression [30]. Further research denoted that the profound presence of protein activity such as proliferating cell nuclear antigen (PCNA) including the disinclination of apoptotic events, by an assortment of metabolic paths, is frequently correlated with the disease prognosis [31]. In conjunction, uncontrolled expressions of protein such as p53, Bax (BcL-2 associated X protein), BcL-2 (B cell lymphoma 2), VEGF (vascular endothelial growth factor), and mTOR (mammalian target of rapamycin) have been found to correspond with the development and growth of colon cancer [32–34]. Furthermore, the Akt/mTOR signaling axis plays a crucial role in the proliferation, resistance to apoptosis, angiogenesis, and metastasis, and that is directed to the development and prolongation of colorectal cancer [35]. Activation of Akt by phosphorylation at ser473 and Thr308 activates several downstream targets including mTOR [36]. Downregulation of mTOR signaling occurs in many types of human tumors including colorectal carcinoma [35]. mTOR is associated with Raptor (mTORC1 complex) to phosphorylate to p70S6K, which further phosphorylates 4E-BP1 that leads to increased cell proliferation [37]. mTOR further associates with Rictor (mTORC2 complex) and functions in a feedback loop and promotes phosphorylates that activate Akt at ser473 [36]. A number of research studies have demonstrated that apoptosis (programmed cell death) is a key procedure to destroy the tumor cells by chemotherapeutic agents [38]. And caspase gives rise to active signaling molecules to propagate the apoptotic process. They are classified by their mechanism of action which includes initiator caspases (caspase-8 and caspase-9) and executioner caspases (caspase-3, caspase-6, and caspase-7) [39]. Caspase-3, a major effector caspase in apoptotic pathways, is an inactive 32 kDa proenzyme. It further cleaves at an aspartate residue to yield a p12 and p17 subunit to form the active caspase-3 enzyme (cleaved caspase-3) [40], which is responsible for morphological and biochemical changes in apoptosis [41].

A widely used compound to promote colorectal carcinogenesis in laboratory rodents is 1,2-dimethylhydrazine (DMH). Dextran sulfate sodium (DSS) is supplied to animals in drinkable water besides DMH to synergize colitis formation as DSS administration causing acute murine colitis comparable to that of ulcerative colitis in humans. As far as we are aware, the ruthenium-phloretin complex is a newfangled entity that is yet to be evaluated for its biological properties. We have synthesized and identified the complex of ruthenium and phloretin throughout this research and assessed its antioxidant interaction and DNA-binding capabilities. Furthermore, the current study explores the chemotherapeutic potential of this unique complex in a HT-29 cell line along with a known-documented prototype of DMH and DSS-mediated colon cancer.

## 2. Materials and Methods

### 2.1. Chemicals, Reagents, and Antibodies

The chemicals, reagents, and antibodies are the following: phloretin and ruthenium (III) chloride hydrate, DPPH, penicillin, TPTZ, ABTS, CT-DNA (calf thymus DNA), foetal bovine serum (FBS), sodium pyruvate, MTT, Annexin V, propidium iodide (PI) (procured from Sigma-Aldrich Chemical Co.), DMH (dimethylhydrazine), streptomycin, DSS (dextran sodium sulfate), insulin, L-glutamine, streptavidin peroxidase, 3,3′-diaminobenzidine (DAB), and proteinase K. Antibodies specific for p53, PCNA, pro- and active caspase-3, Akt1, phospho Akt, mTOR, phospho mTOR, and VEGF (627501) were acquired from BioLegend (San Diego, CA, USA). Anti-rat antibody for Bcl-2, Bax, beta catenin, NF-*κ*B, and goat anti-rabbit IgG secondary antibody were bought from Genetex, Inc. (Irvine, CA, USA). An immunohistochemistry kit was acquired from Biovision, Inc. (Milpitas, CA, USA), and an apoptosis detection kit was purchased from Takara Bio Inc. (Shiga, Japan).

### 2.2. Synthesis of Ruthenium-Phloretin Complex

6.4 g (18.6 mmol) of phloretin was added to 100 ml of methanol while stirring continuously, and the solution was poured dropwise to 2.48 g (9.2 mmol) of ruthenium chloride dissolved in 50 ml of methanol. The solution was stirred continuously for 48 hours by the help of a magnetic stirrer, and after mixing, the reaction mixture was dried in a vacuum using silica gel beads. The dark green colored ruthenium-phloretin complex has been obtained, which was found to be soluble in methanol and dimethyl sulfoxide (DMSO).

### 2.3. Characterization of Ruthenium-Phloretin Complex

Recording of infrared spectra using FT-IR spectroscopy (ALPHA-T, Bruker, Rheinstetten, Germany) in the range 400-4000 cm^−1^ confirmed the structure of the complex. A UV spectrophotometer (UV-1800 Shimadzu) was used to observe the UV-visible spectroscopy of both complex and free phloretin. Electron Ionization Tandem Mass Spectrometry (ESI-MS) technique was used to register the mass spectrometry of the complex, and ^1^H NMR was conducted using a Bruker-Avance-500 MHz. X'Pert Pro XRD (PANalytical) was used to analyze the diffractogram by X-ray study of newly formed compound. Eventually, a scanning-oriented electron microscope (JEOL MAKE, UK, JSM6360) was used to capture the morphological structure of the complex at various magnifications.

### 2.4. In Vitro Antioxidant Properties

FRAP, DPPH, and ABTS method were utilized to discover the in vitro antioxidant properties of the ruthenium-phloretin complex. The reduction of Fe^3+^ ions by the ruthenium-phloretin complex was observed as per Benzie and Strain's method [42]. The process identified by Dolatabadi et al. was followed to determine the DPPH reducing capacity of the complex [43]. The radical scavenging activity percentage was measured as

(1) $$\begin{array}{c} \text{RSA}\%=\frac{100\left(A_{c}-A_{s}\right)}{A_{c}}, \end{array}$$

where *A*_*c*_ denotes absorbance of DPPH and *A*_*s*_ denotes the absorbance of the complex at 517 nm. The method of Pennycooke et al. was used to determine the radical scavenging activity of the complex [44]. The following formula has been used to calculate radical scavenging behavior (RSA):

(2) $$\begin{array}{c} \text{RSA}\%=\frac{1-A_{f}}{A_{0}}\times 100, \end{array}$$

where the absorbance *A*_0_ is that of the unrestrained radical cation and *A*_*f*_ is the absorbance noted 10-12 minutes after the sample addition.

### 2.5. DNA Binding Study

Intercalation of CT-DNA with the compound was determined using a UV-visible spectrophotometer (UV-1800 Shimadzu), based on the method reported by Dehghan et al. [45]. The intrinsic binding constant was calculated as

(3) $$\begin{array}{c} \frac{\text{DNA}}{\varepsilon_{\text{a}}-\varepsilon_{\text{f}}}=\frac{\text{DNA}}{\varepsilon_{\text{b}}-\varepsilon_{\text{f}}}+\frac{1}{K_{\text{b}}\left(\varepsilon_{\text{b}}-\varepsilon_{\text{f}}\right)}, \end{array}$$

where DNA represents the number of base pairing of DNA, *ε*_a_ represents the extinction coefficient (*A*_obs_/Ru) factor, *ε*_f_ is the free drug-related extinction coefficient, and *ε*_b_ represents bound drug-associated extinction coefficient, and the complex-associated calibration curve is derived from *ε*_f_ in the aqueous solution. *ε*_a_ represents the ratio of recorded absorbance to concentration of the complex by Beer's law.

### 2.6. In Vitro Study

#### 2.6.1. Cell Culture

The HT-29 cell line for human colon adenocarcinoma was collected from the National Center for Cell Science (Pune, India). The obtained cells were cultured and maintained in Dulbecco's modified Eagle's medium augmented with 10% FBS and antibiotics at 5% CO_2_, 95% air, and 37°C.

#### 2.6.2. Cytotoxicity Assay

A 96-well plate was taken, and 200 *μ*l of cell suspension was seeded in each well at approximately 20,000 cells per well. After being incubated for 24 hours, each plate was treated with different concentrations (12.5, 25, 50, 100, and 200 *μ*M) of the Ru-phloretin complex. Then, the cells were allowed to grow for 12/24 hours at 37°C fortified with a 5% CO_2_ atmosphere and a complete growth medium. Each well was supplemented with an MTT reagent (0.5 mg/ml), and the plates were finally incubated at 37°C for 3 hours. Lastly, the MTT reagent was tossed out and 100 *μ*l of DMSO was supplemented to each plate. The ELISA plate reader was employed to record the absorbance of individual wells at 570 nm and 630 nm wavelengths. The following equation has been used to calculate the cell viability:

(4) $$\begin{array}{c} \%\text{ viability}=100-\%\text{ of cytotoxicity}. \end{array}$$

#### 2.6.3. Oligonucleosomal Fragmentation

Six-well plates were seeded with HT-29 cells and incubated for 12 hours. The following day, 36.56, 73.13, and 109.69 *μ*M concentrations of Ru-phloretin complex were added to the cells and incubated for 24 hours. The cells were finally collected and fixed in paraformaldehyde (4%) followed by treatment with 0.25% Triton X-100 in TBS meant for 15 min at room temperature. Each well was exposed to 50 *μ*l DAPI (4 mg/ml) for 30 minutes, to be stained. Finally, cells were washed in PBS and stored in darkness at 4°C before being observed by a fluorescence microscope [46].

#### 2.6.4. Detection of Apoptosis by Flow Cytometry

Apoptotic and necrotic cells were detected with an Annexin V-FITC Apoptosis Detection Kit (Sigma Aldrich) as per the instructions of the user manual. 36.56, 73.13, and 109.69 *μ*M of the Ru-phloretin complex were used to treat the HT-29 cells for 24 hours, before being rinsed with PBS. Finally, cells were stained with Annexin V conjugated-FITC and propidium iodide and stored in the dark for 15 minutes. A flow cytometer coupled with Cell Quest software was used for the detection of apoptotic cells.

#### 2.6.5. Cell Cycle Analysis

Different concentrations of the Ru-phloretin complex (36.56, 73.13, and 109.69 *μ*M) were used to treat precultured HT-29 cells for 24 hours. 70% cold aqueous ethanol (*v*/*v*) (2 ml) was used for fixing the cells after the treatment. Then, the cells were centrifuged and rinsed with cold PBS thrice and resuspended in 0.5 ml PBS. 50 *μ*M of RNase A (1 mg/ml in PBS) was added to a 0.5 ml cell sample and incubated for 30 minutes at 37°C. After 5 minutes of gentle stirring at 37°C in the darkness, 50 *μ*M of PI was further applied. A BD FACSCalibur cytometer (Becton Dickinson, Heidelberg, Germany) was used analyze the resuspended cells. 10,000 cells were analyzed per group. The DNA contents were presented with a histogram, and the data assimilated was analyzed with the help of software (ModFit LT 3.2).

#### 2.6.6. SDS-PAGE and Western Blotting

The protein expression levels of VEGF, caspase-3, cleaved caspase-3, Akt, phospho Akt, mTOR, phospho mTOR, and p53 were determined with the help of Western blotting techniques. The cells were collected by trypsinization post treatment with various concentrations of the Ru-phloretin complex. The collected cells were lysed with 50 *μ*l NP40 lysis buffer in ice-cold condition for 1 hour. The cell lysates were centrifuged at 12,000 rpm for 10 minutes at 4°C, and the supernatant was collected. Protein estimation was done by conducting a Bradford assay according to the manufacturer's protocol. An equivalent amount of SDS-loading dye was added to the normalized proteins, and the mixture was boiled for 15 min. Twenty micrograms of protein from each sample was loaded, run on a 10% SDS-PAGE gel, and transferred to 0.25 *μ*m PVDF membranes (Millipore, Bedford, MA, USA). After blocking membranes in 5% nonfat milk in Tris-buffered saline with 0.1% Tween-20 (TBST) for 1 hour at room temperature, the membranes were washed (3x in TBST for 5 min) and incubated with specific primary antibody (antibody dilution according to the product data sheet) at 4°C overnight. The membranes were washed and incubated with HRP-conjugated secondary antibody at a dilution of 1 : 2000 for 1 hour at room temperature. The chemiluminescence of the membrane was measured by an ECL chemiluminescence detection reagent to develop the bands, and the images were captured using ImageJ software [47]. Beta actin was used as a loading control.

### 2.7. In Vivo Study

#### 2.7.1. Experimental Animals and Maintenance

Balb/c mice (25-30 grams), containing animals of both sexes, were used for toxicity study, and for carcinogenicity study, male Wistar rats (80-120 grams) were used. All animals were purchased from Indian IICB, Kolkata, India. Animals were exposed to a light/dark period of 12 hours, with humidity at approximately 50 to 58 per cent and a temperature at 22°C. They were housed in polypropylene cages and given modified pellet diets [48] along with clean drinking water. Animals were accustomed under the above conditions for 10 days prior to the beginning of experiments. The IAEC (Institutional Animal Ethical Committee), which supervised and controlled the experiments (CPCSEA Regn. No. 1458/PO/a/11/CPCSEA), guidelines were strictly adhered to while performing animal experiments and for their care. All experimental work on animals were approved by the IAEC (Ref number: HCG/Pharmacol/2019/05), NSHM College of Pharmaceutical Technology.

#### 2.7.2. Acute Toxicity Study

Acute toxicity studies determined the LD_50_ of the Ru-phloretin complex as per the OECD guidelines for assessment of novel compounds, TG 420 (adopted: December 2001). Five groups of six Balb/c mice (three of each sex) were divided into control and test groups. Test groups were administered with 2000, 600, 400, and 200 mg/kg body weight of the complex suspended in double distilled water with CMC, through gastric intubation. Control groups were provided with only 0.5% CMC solution. Following drug administration, the subjects were given free access to regular food and water while signs of toxicity and mortality were observed for the following three days.

#### 2.7.3. Subacute Toxicity Study

The OECD recommendations were adhered to while performing the 28-day repeated dose toxicity study (oral) in mice (1995; No. 407). The control and test groups (doses 25 mg/kg, 50 mg/kg, 100 mg/kg, and 300 mg/kg of Ru-phloretin complex) were divided into five groups of thirty Balb/c mice, comprising six animals in each group (three per sex). Animals were provided with oral treatments daily for 28 consecutive days and finally sacrificed on the 29th day with anesthetic ether vapors > 4.5% in 100% oxygen until the absence of respiration was observed for greater than 1 minute. After euthanasia, the organs like the kidney, liver, stomach, and testis were extracted for further studies.

*(1) Hematological and Serum Biochemical Analysis*. Blood samples obtained from the retroorbital plexus of animals were used for determining various hematological parameters and further analyzed by Medonic CA-620 cell analyzer systems (Boule Medical, Stock-Holm, Sweden). Biochemical parameters were also analyzed from the blood samples gathered from experimental animals after the serum was removed by centrifuging at 3000 rpm for 10 minutes. An automatic biochemistry analyzer/Pentra C400 (Horiba Medical, Kyoto, Japan) was used for the analysis of biochemical parameters [49].

*(2) Histopathological Analysis*. Important organs extracted from the animals on completion of the 28-day toxicity study were washed in alcohol and fixed in paraffin blocks. These blocks were dissected into 5 *μ*m thickness to be placed on the slides. Before staining the tissues with hematoxylin and eosin (H&E), they were deparaffinized and rehydrated. Prepared slides were visualized under a microscope for analysis.

#### 2.7.4. Experimental Design of Carcinogenicity Study

35 male Wistar rats (80-120 gm) were taken and randomly divided into seven groups (5 rats per group). The animals in group I were administered a pseudotoxic dose of carboxymethyl cellulose (0.5%) by oral gavages, after 10 days of acclimatization whereas DMH at the dosage of 30 mg/kg body weight was provided to the rest of the animals via a single intraperitoneal injection. DSS (2%) was supplied in portable water commencing on the seventh from the day of injection and maintained for a week [50]. The experimental layout is denoted as the following:

Group I animals receiving neither carcinogen nor treatment (normal control).
Group II animals receiving only DMH+DSS (positive control).
Group III animals receiving DMH+DSS and accompanied by 50 mg/kg body weight treatment with the Ru-phloretin complex (test group).
Group IV animals receiving DMH+DSS and accompanied by 100 mg/kg body weight treatment with the Ru-phloretin complex (test group).
Group V animals receiving DMH+DSS and accompanied by 200 mg/kg body weight treatment with the Ru-phloretin complex (test group).
Group VI animals receiving DMH+DSS and accompanied by 100 mg/kg body weight treatment of ruthenium (test group).
Group VII animals receiving DMH+DSS and accompanied by 100 mg/kg body weight treatment of phloretin (test group).

The animals were sacrificed following 20 weeks of treatment under ether anesthesia for analysis.

*(1) ACF Counting*. A portion of colon tissue was sliced open and placed evenly on filter paper with the lumen exposed. Samples were fixed on slides with the help of 10% buffered formalin; after 12 hours, the samples were stained with 0.2% methylene blue prepared in PBS and examined through a microscope (4x magnification). ACFs could be distinctively characterized by their microscopically pronounced slit openings with a dense epithelial casing and profound stain. ACF has been reported as the sum total of occurrences per 5 cm^2^ [51].

*(2) Histopathological Analysis of Colon*. Ten random animals were selected from individual collections, and their colon was removed. Before placing on the slides, the tissues were systematically processed and cut into 5 *μ*m thickness. Hematoxylin and eosin (H&E) were used to stain the tissues before further analysis.

*(3) Antioxidant Activity of Colon Tissues*. Collected colon tissues were compressed and homogenized (10% *w*/*v*) in 0.1 M phosphate buffer (pH 7.0). The homogenized mixture was centrifuged for 10 minutes to evaluate the antioxidant activity. The procedure of Jagatheesh et al. was employed to obtain the activity of the supernatant [52]. Catalase activity was carried out in the method illustrated by Sinha and his colleagues [53]. A superoxide dismutase assay was conducted with the technique developed by Awasthi et al. [54]. GPx activity was assessed using the Rotruck et al. method [55].

*(4) Immunohistochemical Analysis*. The obtained colon tissues were preserved in formalin and fixed in paraffin. The tissues were further cut to a thickness of 5 *μ*m and mounted on glass slides and then deparaffinized and immersed in H_2_O_2_. In the next step, tissues were exposed to treatment with goat serum and subsequently exposed to anti-rat Bcl-2, NF-*κ*Β, Bax, and MMP-9 antibodies (1 : 200) and kept at 4°C for the next 12 hours. The slides were finally rinsed with PBS and exposed to biotinylated HRP-conjugated secondary antibody for 30 minutes. The streptavidin biotin HRP complex-treated slides were further treated with DAB chromogen and stained with hematoxylin. The labeling index was expressed as the fraction of stained cells by the total number of cells counted [56].

*(5) Cell Proliferating Assay*. Deparaffinized and rehydrated with graded alcohol, sections of poly-L-lysine-coated tissue were submerged in H_2_O_2_ (3%) accompanied by 1-hour goat serum incubation. Tissue segments were rinsed in PBS and incubated overnight at 4°C with anti-rat PCNA antibody. Tissues had been stained with DAB as chromogen with hematoxylin as counterstain [57].

*(6) Apoptosis Assay by Tunnel Method*. An apoptosis assay was conducted by the using the In Situ Apoptosis Detection Kit by Takara Bio Inc. (MK500) as per the manufacturer's protocol, developed with DAB as chromogen and methyl green (Loba Chemie Pvt. Ltd.) as counterstain. The sections were cleaned, desiccated, and mounted. Identification of apoptotic cells was done by observing the reddish brown-stained nuclei [57].

*(7) Determination of Labeling and Apoptotic Index*. The ratio of the percentage of PCNA-stained nuclei to the overall number of cells gave the labeling index (LI). The apoptotic index (AI) has been determined as the % of stained cells by the total cells observed.

### 2.8. Statistical Analysis

Data were reported as the mean ± standard error mean (SEM). Statistical testing was conducted using *t*-test along with ANOVA assisted by GraphPad Prism, accompanied by Dunnet's *t*-test; the alterations were evaluated as statistically relevant at *p* < 0.05.

## 3. Results

### 3.1. Instrumental Analysis

**Figure 1:**
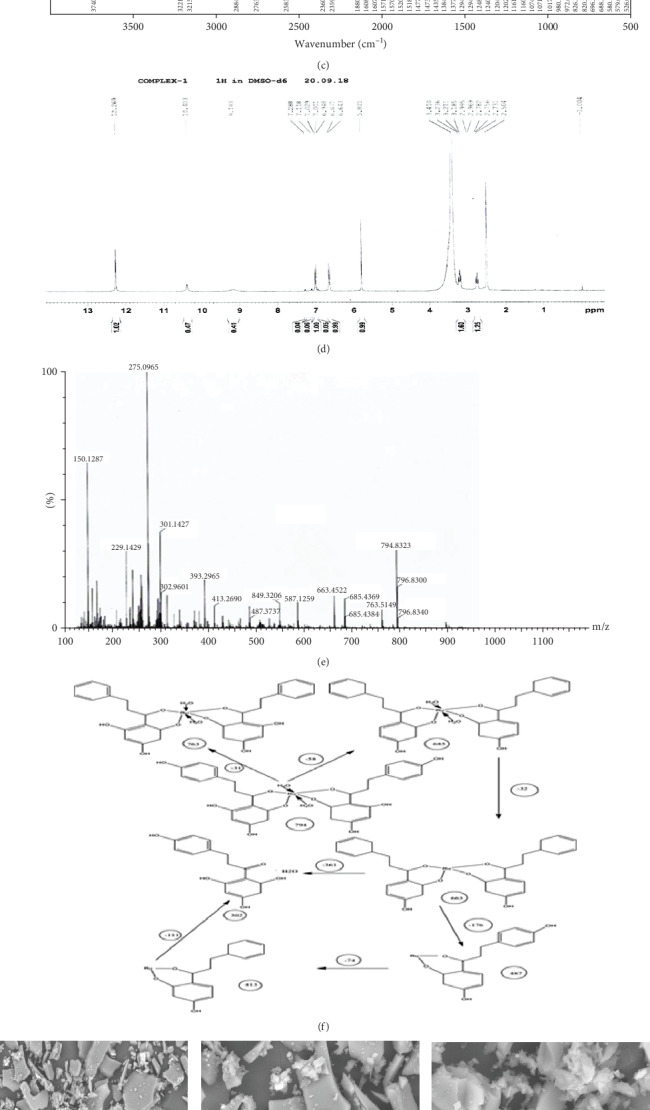
(a) Ruthenium-phloretin complex. (b) UV-visible spectrum of phloretin and ruthenium-phloretin compound. (c) FTIR spectra of phloretin and ruthenium-phloretin complex. (d) NMR spectra of ruthenium-phloretin complex. (e) Mass spectroscopy of ruthenium-phloretin complex. (f) Fragmentation mechanism of the ruthenium-phloretin complex. SEM of the complex at (g) 200 *μ*m, (h) 100 *μ*m, and (i) 50 *μ*m. (j) X-ray diffractogram of ruthenium-phloretin complex.

UV-visible spectroscopy did not show any significant changes in the absorption spectra of phloretin and ruthenium-phloretin complex. The ruthenium-phloretin complex slightly showed charge transfer transitions. Both phloretin and complex showed strong absorption bands at 280430 nm (Figure 1(b)). The ruthenium-phloretin complex exhibited only charge transfer transitions, from the ligand (RIF) to the metal. Therefore, no d-d transitions are expected for Ru (III) complexes. The ruthenium-phloretin complex and free phloretin FTIR spectra were documented to establish the coordinating sites and binding characteristics of the complex as shown in Figure 1(c) and assessed in Table 1.The v(O-H) broad bands appeared at 3221.01 cm^−1^ and 3215.23 cm^−1^ in the IR spectrum of phloretin and ruthenium-phloretin complex showing the presence of water molecules. The v(C=C) stretching occurred at 1571.75 cm^−1^ for the complex. The v(C=O) stretching occurred at 1384.23 cm^−1^ and 1248 cm^−1^ for phloretin whereas for the complex, it is seen at 1377.28 cm^−1^ and 1240.53 cm^−1^ correspondingly. The v(COH) bond shifted from 980.13 cm^−1^ to 972.67 cm^−1^ for the ruthenium-phloretin complex. The characteristic band for the ruthenium-phloretin complex was seen at 614.22 cm^−1^ which was absent in free phloretin. These results indicate that maybe the OH group present in phloretin can coordinate with ruthenium to form a coordination complex.  Table 2 shows the chemical transition of ^1^H NMR spectrum of the complex and the unbound ligand. The observations reveal the omission of 3-OH and 9-OH protons in the spectra of the complex, signifying that ruthenium confiscates two protons from the flavonoid phloretin upon complexation, while the other protons were found to be slightly shifted and are intramolecularly bonded (Figure 1(d)). The above evidence suggests that the chelation occurred via the 3-OH and 9-OH functional groups of the ligand. The mass spectroscopy of the ruthenium-phloretin complex is shown in Figure 1(e); the base signal at *m*/*z* 275 was of free phloretin whereas *m*/*z* 302 was of phloretin+two water molecules. The signal at *m*/*z* 487 was seen for one phloretin+ruthenium each. The molecular peak for the ruthenium-phloretin complex was seen at *m*/*z* 794 where two phloretin+one ruthenium+two water molecules coordinated to form the complex. The fragmentation is depicted in Figure 1(f). Figures 1(g)–1(i) exhibit the surface structural arrangement of the ruthenium-phloretin complex, evaluated by SEM which denotes crystalline in nature and asymmetrical shape of the complex. The X-ray diffraction study of the complex indicates multiple distinctive sharp peaks which occurred at different diffraction angles attributable to its recognizable crystalline structure (Figure 1(j)).

### 3.2. Ruthenium-Phloretin Complex Scavenges DPPH, FRAP, and ABTS Radicals

**Figure 2:**
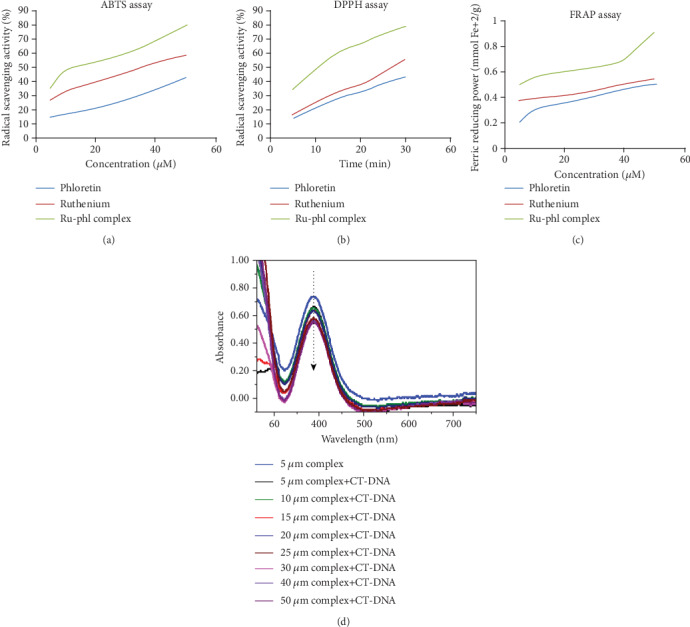
Assessment of antioxidant status: (a) antioxidant property of the ruthenium-phloretin complex by ABTS procedure, (b) DPPH method, (c) FRAP procedure, and (d) absorbance spectrum of CT-DNA in complexation with ruthenium-phloretin.

Figure 2(a) displays the scavenging of radicals by free phloretin and ruthenium-phloretin complex by ABTS. It was observed that by varying the concentrations of the complex, the absorption of activated ABTS at 734 nm dropped significantly. The complex was able to scavenge free radical more efficiently in the presence of ABTS as compared to free phloretin. The antioxidant behavior of the ligand is considerably correlated with their molecular structure.

The complex's strong antioxidant activity could be attributed to the hydroxyl functional group and their capacity to contribute hydrogen atoms which has been considerably improved after the complexation with ruthenium.

Figure 2(b) illustrated the scavenging of radicals by phloretin and ruthenium-phloretin complex, where the plot suggested that phloretin scavenged free radical to about 43% while the complex scavenged to about 79%. Thus, the complex represented better inhibitory effect compared to the phloretin. These are mainly attributed to synergistic effects of the ruthenium-phloretin complex. The radical-sensitive Ru-O bond was introduced to the ruthenium-phloretin complex, which collectively enhances the bioactivities of both ruthenium and phloretin.

The availability of Fe^+3^-TPTZ was observed in the presence of phloretin and ruthenium-phloretin complex at 593 nm by altering the difference absorbance, for a 10 min interaction, of the complex and phloretin in the FRAP solution. Declination in the absorbance was in proportion in aspect to the antioxidant range of the complex and the ligand.  Figure 2(c) reaffirms that the complex possesses a greater antioxidant power than free phloretin. These observations denote that the phloretin and ruthenium-phloretin complex can donate protons and are capable of terminating a chain reaction. Moreover, metal chelation augments the transfer of electrons from phloretin and hence improves the redox potential of the ruthenium-phloretin complex.

### 3.3. Ruthenium-Phloretin Complex Binds with CT-DNA

The absorption spectrum of the complexes in the presence of CT-DNA (5 *μ*Μ) is shown in Figure 2(d). Upon addition of increasing amounts of the complex to CT-DNA, a decrease in the absorption intensity (hypochromism) of the absorption peak is observed. After increasing the concentration of the complex to the DNA, the intensity changes can be identified within the intraligand transition band at 383 nm. These spectral characteristics reveal that the complex interacted with DNA via stacking interaction between the chromophore of the ligand through the intercalative mode and the base pairs of DNAs.

### 3.4. In Vitro Study

#### 3.4.1. Inhibition of HT-29 Cell Proliferation by Ruthenium-Phloretin Complex

**Figure 3:**
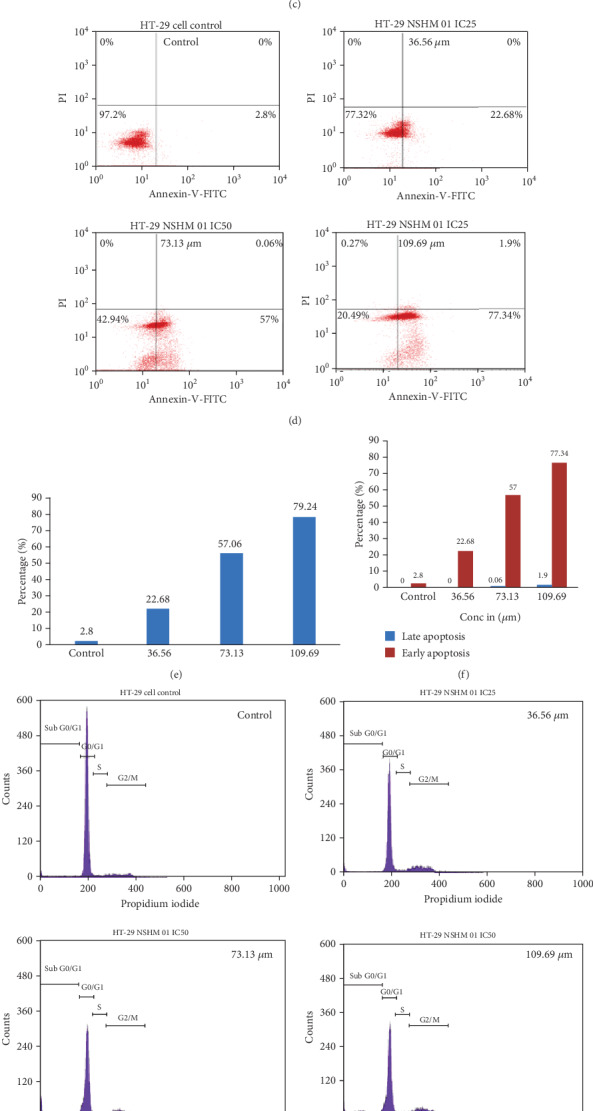
HT-29 cell viability of ruthenium-phloretin complex at (a) 12 hours and (b) 24 hours. (c) DAPI staining of colon carcinoma cells treated with ruthenium-phloretin complex. (d) Flow cytometric analysis of HT-29 cells for detection of apoptotic events after treatment with ruthenium-phloretin complex. (e) Proportion of apoptotic cells against concentration. (f) Percentage of apoptotic cells in early and late apoptosis stages. (g) Cell cycle analysis of HT-29 cells following exposure to ruthenium-phloretin complex. (h) Various phases of cell cycle analysis of HT-29 cells. (i) Analysis of Western blot to determine the expressions of p53, caspase-3, cleaved caspase-3, Akt, phospho Akt, VEGF, mTOR, and phospho mTOR in HT-29 cells.

Figures 3(a) and 3(b) denote the effect of the ruthenium-phloretin complex on the growth of HT-29 cells assessed by MTT. The cell viability of the complex at 12.5, 25, 50, 100, and 200 *μ*M after 24 hours was found to be 90.17%, 75.28%, 59.61%, 45.50%, and 22.95%, respectively. It can thus be assumed that the development of HT-29 cells decreased markedly in a dose-dependent manner following ruthenium-phloretin complex treatment, with the highest inhibition at 77.05% after treatment with 200 *μ*M of the ruthenium-phloretin complex after 24 hours.

#### 3.4.2. Ruthenium-Phloretin Complex Promotes Apoptosis of HT-29 Cells

Treatment of HT-29 cells with 36.56, 73.13, and 109.69 *μ*M of the ruthenium-phloretin complex for 24 hours evidently reduced the number of viable cells. As shown in Figure 3(c), the untreated control cells maintained their unique shape and their nuclei were homogeneously stained with a dull blue color. Treated cells exhibited more intense blue fluorescence as compared to the untreated cells. This more intense staining may be due to the presence of highly condensed chromatin or because of cell cycle arrest.

To further confirm the apoptosis-inducing activity of the ruthenium-phloretin complex, HT-29 cells were subjected to Annexin V-FITC/PI staining with flow cytometry. The analysis of the results revealed that the percentages of apoptotic cell are 22.68%, 57.06%, and 79.24% following treatment with 36.56, 73.13, and 109.69 *μ*M of the complex (Figures 3(d) and 3(e)). In addition, a dose-dependent improvement was also identified in the areas of early apoptotic cells in 36.56, 73.13, and 109.69 *μ*M of the complex-treated cells after 24 hours (Figure 3(f)).

#### 3.4.3. Ruthenium-Phloretin Complex Promotes Cell Cycle Arrest in HT-29 Cells

The inhibition of cell proliferation could be the result of the inception of apoptosis that is mediated by cell cycle arrest. Therefore, the cell cycle distribution in the HT-29 cells treated with the ruthenium-phloretin complex was further analyzed. A decreased percentage of cells in the G0/G1 phase together with a slight increase in the S phase was observed to occur in a dose-dependent manner (Figures 3(g) and 3(h)), while the complex interrupts the cell cycle G2/M phase in a dose-dependent way. When exposed to 36.56, 73.13, and 109.69 *μ*M of the complex after 24 hours, the cell population of HT-29 cells in the G0/G1 phase was 59.64%, 58.54%, and 54.98% at 24 hours (Figure 3(h)). As the number of cells in the G0/G1 phase decreases with the increase of the drug concentration, we can conclude that the complex arrests the cell cycle G0/G1 process in a dose-dependent way.

#### 3.4.4. Ruthenium-Phloretin Complex Alters the Illustration of p53, Caspase-3, Akt1, mTOR, and VEGF in HT-29 Cells

To explore the pattern of ruthenium-phloretin-induced apoptosis of HT-29 cells, the expressions of various proteins involved were evaluated using Western blotting. After 24-hour treatment with 36.56, 73.13, and 109.69 *μ*M of the compound, a concentration-dependent decline of Akt1, phospho Akt, mTOR, phospho mTOR, and VEGF was recorded. Consequently, an increase in the p53 and pro- and active caspase-3 expressions was observed in HT-29 cells following complex administration in a dose-dependent manner.

### 3.5. In Vivo Study

#### 3.5.1. Toxicity Study

*(1) Acute and Subacute Toxicity Study*. The LD_50_ dose was found to be 400 mg/kg of the ruthenium-phloretin complex. Following the LD_50_ dose assessment, 25, 50, 100, and 300 mg/kg were selected as the subacute toxic doses. During subacute toxicity analysis (28 days), no treatment-related fatalities were reported in animals treated with 25, 50, 100, or 300 mg/kg of the complex.

*(2) Hematological and Serum Biochemical Analysis*. Tables 3–6 show the hematological and serum biochemical profile of the treated and control groups. WBC and RBC amounts were significantly augmented in ruthenium-phloretin complex (300 mg/kg)-treated groups contrasting with control animals. At the completion of the 28-day subacute toxicity test, ALT, AST, and ALP were significantly higher than the control group at 300 mg/kg (*p* < 0.05). Glucose and BUN were also significantly (*p* < 0.05) altered at 300 mg/kg compound-treated animals. Therefore, the 300 mg/kg dose of the complex caused toxicity in the laboratory animals to some degree and was therefore not regarded as a dose for ensuing study.

**Figure 4:**
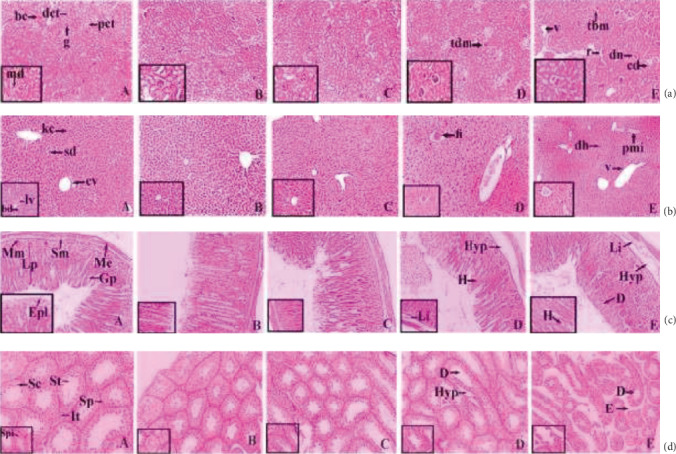
(a) Histopathology of kidney of Balb/c mice: (A) normal tissue showing glomerulus (g), proximal convoluted tubule (pct), macula densa (md), Bowman's capsule (bc), and distal convoluted tubule (dct); (B) kidney tissue exposed to 25 mg/kg complex; (C) kidney tissue exposed to 50 mg/kg complex; (D) kidney tissue exposed to 200 mg/kg complex; (E) kidney tissue exposed to 600 mg/kg complex showing capsular membrane thickening (tbm), desquamated nuclei (dn), ruptures (r), and vacuolization. (b) Histopathological representation of liver of Balb/c mice: (A) normal control expressing the central vein (cv), bile duct (bd), sinusoidal dilation (sd), Kupffer cell (kc), and lymph vessel (lv); (B and C) kidney tissue exposed to 25 mg/kg and 50 mg/kg of complex; (D and E) kidney tissue exposed to 100 and 300 mg/kg complex exhibited periportal mononuclear infiltration (pmi), disintegration of hepatocyte (dh), and inflammation in focal region (fi). (c) Histopathological representation of the stomach of Balb/c mice: (A) normal control exhibited muscularis externa (me), submucosa (sm), muscularis mucosa (mm), lamina propia (lp), pit of the gastric region (gp), and lining of the epithelial region (epl); (B and C) stomach tissue exposed to 25 mg/kg and 50 mg/kg of complex; (D and E) stomach tissue exposed to 100 and 300 mg/kg complex showing hemorrhages (h) between villus, hyperplasia (hyp), and infiltration of leukocytes (Li). (d) Histopathological representation of the testis of Balb/c mice: (A) normal control exhibiting Sertoli cell (sc), spermatogonia (Sp), and seminiferous tubule (St), and tissues of interstitial region (It) are seen within the tubular lumen; (B and C) testis exposed to 25 mg/kg and 50 mg/kg of complex; (D and E) testis exposed to 100 and 300 mg/kg complex exhibited edema in interstitial region (E), deterioration of seminiferous tubule (D), and hyperplasia (Hyp). H&E: 10x magnification (inset 40x).

*(3) Histopathology*. Histopathology of the kidney (Figure 4(a), A) of the control group exhibited a normal structural architectural organization. The foremost morphological variations were observed at the 300 mg/kg dose (Figure 4(a), E). 25 and 50 mg/kg doses did not denote any major abnormalities in the animals (Figure 4(a), B and C), whereas minor thickening of Bowman's capsule was detected in the mice treated with doses of 100 mg/kg complex (Figure 4(a), D). At 600 mg/kg vacuolization (v), pyknotic nucleus (pn), cytoplasmic debris (cd), capsular membrane thickening (tm), and sclerosis at nodes (n) were observed. The histopathology of the liver (Figure 4(b) , A) denoted standard hepatic structures in the control group whereas maximum doses (300 mg/kg) of the ruthenium-phloretin complex denoted focal inflammation (fi), degeneration of hepatocytes (d), and periportal mononuclear infiltrates (pmi) (Figure 4(b), E). 25, 50, and 100 mg/kg dose-administered animals did not exhibit any vital deformity (Figure 4(b), B–D). Figure 4(c) reveals the microscopic assessment of the stomach, where the 300 mg/kg dose of the ruthenium-phloretin complex showed the congestion (c), hemorrhages (h), and hyperplasia of the gastric glandular zone (hyp) (Figure 4(c), E). But at the lower dose level (25 mg/kg, 50 mg/kg, and 100 mg/kg), histopathological variations were not observed (Figure 4(c), B–D). Figure 4(d) reveals the microscopic assessment of the testis, where 300 mg/kg (Figure 4(d), E) of the ruthenium-phloretin complex-treated animals exhibited degeneration in seminiferous tubules (D) and edema in interstitial tissues (E), and at 100 mg/kg (Figure 4(d), D), degeneration (D) and hyperplasia (hyp) were noted. But at the lower dose level (25 mg/kg and 50 mg/kg), no histopathological alterations were observed (Figure 4(d), B and C).

#### 3.5.2. Carcinogenicity Study

**Figure 5:**
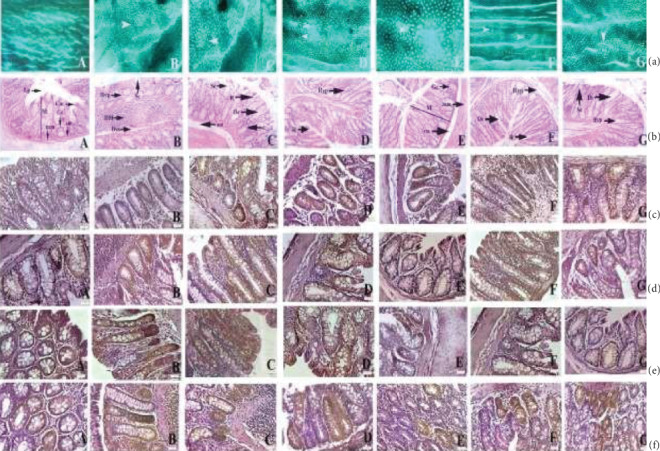
(a) Assessment of ACF on colonic tissue: (A) normal control group; (B) DMH and DSS-treated control group; (C) 50 mg/kg of ruthenium-phloretin-treated group; (D and E) 100 and 200 mg/kg of ruthenium-phloretin complex treated, respectively; (F) ruthenium 100 mg/kg; and (G) phloretin 100 mg/kg served animals. Denotion of the crypts done with white arrows. (b) Histological analysis of rat colonic mucosa at 10x magnification (inset 40x): (A) normal control rat showing the submucosal layer (sm), mucosal layer (M), lamina propia (Lp), muscularis mucosa (mm), columnar absorptive cells (Cac), and goblet cell (Gc); (B) hyperplasia of mucosa observed in DMH and DSS-treated rat colon (Hyp), mucin depletion (DM), epithelial cell sloughing (Se), and dysplasia (Dys); (C) DMH and DSS and 50 mg/kg ruthenium-phloretin-treated animals exhibited sloughing of epithelial cells (Se), crypts (c), dilation of column (Dc), and occasional rupture (R); (D) DMH and DSS and 100 mg/kg ruthenium-phloretin-treated animals showing hyperplastic lesion of mucosa (Hyp) and attenuation of goblet cells (D); (E) DMH+DSS+200 mg/kg ruthenium-phloretin-treated animals exhibited practically normal histological structure; (F and G) DMH and 100 mg/kg ruthenium and DSS and DMH treatment with DSS and 100 mg/kg phloretin exposed animals exhibited hyperplasia (Hyp), dysplasia (Dys), sloughing of epithelial cells (Se), dilation of column (Dc), and occasional rupture (R). The immunohistochemical analysis of (c) Bax, (d) Bcl-2, (e) NF-*κ*B, and (f) MMP-9 expressions in the colon tissues of different groups of rats: (A) the normal control; (B) carcinogen control; (C) 50 mg/kg of complex treated; (D and E) 100 and 200 mg/kg complex treated; (F and G) 100 mg/kg ruthenium and 100 mg/kg phloretin-treated animals. All images at 40x. Scale bars represent 100 *μ*m in all images.

*(1) Ruthenium-Phloretin Treatment Suppressed the Formation of Aberrant Crypt Foci in Rats*. To evaluate the effect of ruthenium-phloretin on suppressing colon carcinogenesis, ACF was assessed as a biomarker to detect early-stage colon cancer in rats. The incidence of ACF on the colon mucosa was analyzed with methylene blue staining immediately after the sacrifice of animals, and the data are depicted in Figure 5(a) and Table 7. ACF is typically characterized as crypts with eminent sizes, altered luminal epithelia, and apparent pericryptal zones. The incidence of ACF was noticeably decreased in the groups treated with the complex as compared to the carcinogen control group (Figure 5(a), B). Compared to the carcinogen control groups, a substantial decrease in the multiplication of ACF was discernible in the groups treated with 50 mg/kg and 100 mg/kg of the complex, respectively (Figure 5(a), C and D), but the greatest reduction was most noticeable in the groups treated with the highest dose (200 mg/kg) of the complex (Figure 5(a), E) (*p* < 0.01). No substantial decrement in ACF multiplicity was noted in the ruthenium and phloretin-treated groups (Figure 5(a), F and G).

*(2) Ruthenium-Phloretin Treatment Altered the Colonic Mucosa in Rats*. After 20 weeks of treatment, colonic tissues were examined histopathologically at 40x by H&E staining where the pristine cylindrical shape and typical architecture of crypts are visible in the control group (Figure 5(b), A). However, in the carcinogen control group (Figure 5(b), B), the correlation between crypt architecture and stromal tissues has been deformed due to dysplasia and enlarged nuclei. The dysplastic tissue is demarcated by a mucosal proliferation and promotes mitosis with diminution of goblet cells and completes exhaustion of mucin. Immense hyperplasia can also be identified by the number of deeply stained nuclei due to the presence of highly proliferative cells. The coadministration of the ruthenium-phloretin complex in 50 and 100 mg/kg-treated groups (Figure 5(b), C and D) significantly corrected these carcinogenic changes. The histopathological architecture of the colonic tissue of 200 mg/kg-treated rats (Figure 5(b), E) resembles that of the control animals. However, no significant improvement was noted in the tissues of animals supplemented with ruthenium and phloretin 100 mg/kg (Figure 5(b), F and G).

**Figure 6:**
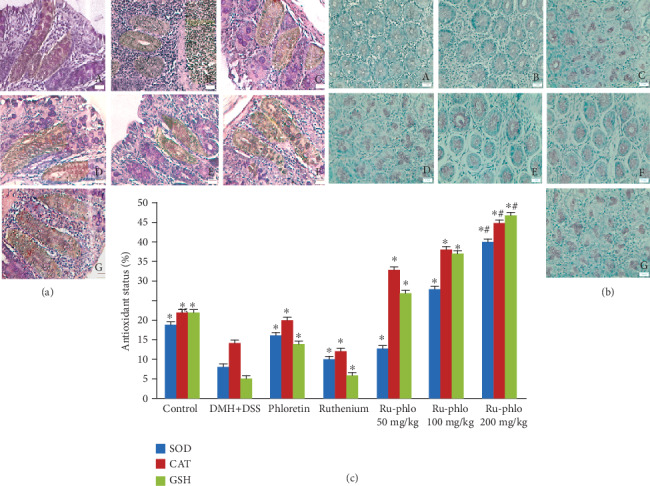
(a) Immunohistochemistry of PCNA in rat colonic mucosa: (A) the normal control; (B) carcinogen control; (C) 50 mg/kg of complex treated; (D and E) 100 and 200 mg/kg complex treated; (F and G) 100 mg/kg ruthenium and 100 mg/kg phloretin-treated groups. All images at 40x. (b) TUNEL assay of apoptotic; (A) the normal control; (B) carcinogen control; (C) 50 mg/kg of complex treated; (D and E) 100 and 300 mg/kg complex treated; (F and G) 100 mg/kg ruthenium and 100 mg/kg phloretin-treated groups. All images at 40x. (c) Effect of ruthenium-phloretin complex on in vivo antioxidant enzymes SOD (superoxide dismutase), CAT (catalase), and GST (glutathione). ^∗^*p* < 0.05 represents the value as contrasted to the carcinogen control; ^#^*p* < 0.01 represents the value as contrasted to the carcinogen control. Scale bars represent 100 *μ*m in all images.

*(3) Antioxidant Assessment of Ruthenium-Phloretin Complex in Colon Tissue*. The quantity of SOD, CAT, and reduced glutathione plunged considerably in the carcinogen control animals (Figure 6(c)). However, the highest dose of the complex (200 mg/kg) exhibited a prominent upgrade in the SOD, CAT, and glutathione levels of the colon tissues with respect to carcinogen control and other treated groups (*p* < 0.01).

*(4) Regulation of Bax, Bcl-2, NF-κB, and MMP-9 Pathways by Ruthenium-Phloretin Treatment*. To delineate the effect of ruthenium-phloretin treatment on the rat colonic mucosa, the presence of cellular biomarkers like Bax, Bcl-2, NF-*κ*B, and MMP-9 was assessed by the immunohistochemical staining techniques (Figure 5 and Table 8). It was observed that DMH and DSS administration downregulated in a significant way the levels of Bax (Figure 5(c), B); however, an increment of Bcl-2 (Figure 5(d), B), NF-*κ*Β (Figure 5(e), B), and MMP-9 (Figure 5(f), B) levels were perceived, related to the control animals (Figures 5(c), A; 5(d), A; 5(e), A; and 5(f), A) (*p* < 0.05). The Bax illustration (Figure 5(c), C–E) was significantly increased in the ruthenium-phloretin complex-treated animals, whereas the revelation of Bcl-2, NF-*κ*Β, and MMP-9 was reduced in a significant manner (Figures 5(d), C–E; 5(e), C–E; and 5(f), C–E) especially with respect to carcinogen-treated animals (*p* < 0.05). The dose of 200 mg/kg body weight of the ruthenium-phloretin complex was immensely powerful in amplifying the appearance of Bax and concurrently declined the Bcl-2, NF-*κ*Β, and MMP-9 protein levels (*p* < 0.01) relative to the carcinogen-treated animals. The appearance of the aforementioned biomarkers orients us to perceive that the complex actuates apoptosis and furthermore restricts cell cycle to adequately restrict disease progression.

*(5) Repression of PCNA by Ruthenium-Phloretin Complex*. The efficacy of the ruthenium-phloretin compound in the proliferation of colon tissues is designated in Figure 6(a). The LI (labeling index) is calculated as the fraction of PCNA-labeled cells represented in Table 9. A significant rise in the PCNA-LI values was observed in carcinogen control animals (Figure 6(a), B); however, a considerable reduction in the PCNA-LI index could be found in the maximum dose of the ruthenium-phloretin complex-treated animals (*p* < 0.01) (Figure 6(a), E). Ruthenium-phloretin at 100 mg/kg-treated groups (Figure 6(a), D) also showed a decrease in the PCNA-LI values (*p* < 0.05) while the rest of the animals did not show any noteworthy alteration at levels of PCNA in comparison to the carcinogen-treated animals.

*(6) Ruthenium-Phloretin Complex Promotes Apoptosis*. The TUNNEL assay was carried out to visualize the result of ruthenium-phloretin treatment on apoptosis in colon cancer (Figure 6(b)). Apoptosis causes the nuclear DNA to rupture into smaller fragments yielding DNA strand breaks, which can be detected by the brown stains, formed by DAB chromogen. The TUNEL-positive cells undergoing apoptosis were very few in the case of the carcinogen control group (Figure 6(b), B), whereas the TUNEL-positive cells of ruthenium-phloretin-treated animals increased significantly (Figure 6(b), C–E). In an area with almost 700 cells, 3 to 5 apoptotic cells were usually observed in the carcinogen control group, which raised to 10-14 cells every 700 cells throughout the 200 mg/kg of the complex-treated animals. AI defines the apoptotic index and is documented in Table 9. Animals receiving 200 mg/kg of the compound denoted a substantial increase in apoptosis when contrasted to the carcinogen control group. The value *R* reflects the ratio of cell proliferation to apoptosis. Cellular proliferation and TUNNEL assessment indicate that perhaps the latest alterations in the microenvironment of the tumor are accompanied by a concurrent boost in cell proliferation as well as minimizing cell death. The value of *R* reaches a peak in the carcinogen control group; however, it declines steadily with the increase in the dose concentration of the complex. By stating all of these assumptions, we can infer that the complex triggers apoptosis and consequently decreases proliferation of the cells in a concentration-controlled fashion.

## 4. Discussion

For a competent module of administration, modern anticancer drugs derived from metal complexes emphasize on inducing apoptosis in cancer cells which provide marked improvements in pharmacological applications [58]. The move was greatly inspired by the development of platinum-based antitumor drugs, but numerous drawbacks such as extreme adverse side effects, drug resistance, accumulation of mutations, and epimutations force us to look for alternative therapies. Allardyce and Dyson mentioned in their journal article that ruthenium a platinum group of metal has many enticing physiological effects [11] and can develop strong chemical bonds with varying electronegativity that enable it to bind to a multitude of biomolecules [59].

Bioflavonoids, a group of polyphenols originating in plant-based diets, are the captivating substitute to metal-based treatment in CRC. Bioflavonoids possess significant anticancer properties due to their potentiality to trigger the apoptosis, metabolic activation, cell proliferation, adhesion of tumor cell, and angiogenesis [60]. In addition, these compounds can chelate to form complexes with a wide range of metal ions and have considerable radical scavenging activities as compared with the flavonoids, alongside having a pivotal role in countering oxidative stress [61]. Phloretin is one such natural product found in plants that exhibits a range of bioactivities such as anticancer, antimicrobial, and antioxidant activities [62]. Phloretin is known to cause apoptotic cell death in the cancer cells of the esophagus [63]. Phloretin can increase cisplatin's potency in treating lung cancer [64] and has been observed to cause apoptosis in human leukemia cells [65]. Being a natural flavonoid (dihydrochalcone), phloretin has almost no cytotoxicity against normal cells. In this present research, we explored the potential impact of the ruthenium-phloretin complex on colon carcinoma in correlation with in vitro and in vivo protocols.

During our analysis, we synthesized the molecules, and further, it has been characterized. We applied different spectroscopic assessments to determine the antioxidant capacity of phloretin before and after the complex formation. Results confirm that the chelation materializes by the 3-OH and 9-OH functional groups of the ligand, and the complex is found crystalline. The study of antioxidant activity revealed that the property of free radical scavenging of phloretin is considerably intensified on subsequent complexation with metal. Therefore, ruthenium facilitates to modify the oxidative capacity of phloretin following complexation by increasing the shifting of electrons from phloretin and hence escalating its redox potential. The reaction of the complex through CT-DNA ensued in a decline in the spectrum of absorption of uncombined DNA, evidence of the phloretin complex intercalated with CT-DNA.

The subsequent module of the study was dedicated to determine the outcome of the ruthenium-phloretin complex on the HT-29 cancer cell line. The MTT assay revealed that the ruthenium-phloretin complex is capable of reducing cellular propagation and initiates apoptosis. Among the most consequential objectives of anticancer drugs is the cell cycle regulation; particularly, the arrest of phases G1 and G2 offers a crucial role in the sequence of the cell cycle [66]. An understanding into the mechanistic approach of the apoptotic induction by the complex was obtained by flow cytometry studies applying Annexin V and PI staining. Furthermore, results showed that a greater proportion of early apoptotic events is marked by ruthenium-phloretin in the HT-29 cancer cells (Figure 3(f)) by detaining the cells in the G0/G1 stage (Figure 3(h)) that consequently leads to cellular death.

The global harmonized program for classifying and marking chemicals includes the recording of the safest dose for a novel cancer entity [67]. Hence, acute and subacute toxicological studies were carried out to assess the LD_50_ value and safe doses of the complex. Furthermore, a cell-oriented reporter assessment has been performed to evaluate the result of ruthenium-phloretin complex treatment on the appearance of VEGF, Akt, phospho Akt, p53, pro- and active caspase-3, phosphor mTOR, and mTOR-related signaling trail. In recent scenarios, the gene mutation in cancer is caused by tumor suppressor gene TP53. Mutation of TP53 and the simultaneous inactivation of p53 suppress tumor cell death and progression [68]. The expressions of PTEN are controlled by cellular p53 levels, which in turn negatively regulate the PI3 kinase pathway that explains the indications from tyrosine kinase receptors to amend the cell-regulated setting [69]. Activation of P13 assisted by PTEN causes the Akt phosphorylation that in turn activates the mTOR pathways kindling tumorigenesis [70]. There is a recent preclinical study with OSI-027, a potent inhibitor of Akt/mTOR which suppressed the growth of colorectal cancer [71]. In addition, Akt and mTOR inhibitors can downregulate leptin-mediated PI3K/Akt/mTOR signaling which influences the colon cancer cells to proliferate and promote apoptosis [72]. Our studies in the manuscript had similar results with ruthenium-phloretin complex effectively, downregulating both Akt and mTOR and their phosphorylated forms. Additionally, mTOR also upregulate cyclins responsible for controlling the action of enzymes essential in the passage of cells all the way through G1-S stages [70]. mTOR is further responsible for the regulation of the angiogenic growth factor similar to VEGF, which is involved towards the progress of tumors in mammary, cervical, ovarian, and gastrointestinal cancers [70]. Besides, p53 promotes the caspase cascade through the intrinsic apoptotic pathway to degenerate several cells derived from proteins in sequence to foster the morphological variations essential in apoptosis [73].

Our experiments aim is to reveal that the ruthenium-phloretin complex operates alongside a unique mechanism by escalating the caspase-3 and p53 proteins and subsiding mTOR, VEGF, and Akt expressions. Hence, apoptosis is modulated through intrinsic trail subsequently by initiating the caspase pathways. Additionally, the complex exhibits the cell cycle arrest by detaining the G0/G1 point. Our results fruitfully demonstrated that the complex treatment profoundly suppressed VEGF, Akt, and mTOR, expressions that could contribute significantly in the prevention of the angiogenic process. Furthermore, our research provides abundant proof that the ruthenium-phloretin complex pursues a p53-dependent apoptotic cell death in colon carcinoma. One of the principal aspects accompanying the cellular apoptotic process is the Bax and Bcl-2 proteins, which are typically associated with Akt and extracellular signal-regulated kinase (ERK) trail [74]. Bax, proapoptotic in nature, is essential for the apoptotic process in regular cells and to inhibit unwarranted proliferation and the prospect of tumorigenesis. Bcl-2 proteins, however, act in the opposing manner by promoting cell survival and suppressing apoptosis [75]. Ruthenium-phloretin treatment downregulates Bcl-2 intensity and enhanced the proapoptotic proteins, such as Bax and caspase-3, thus escalating the Bax/Bcl-2 ratio and encouraging the apoptotic process.

Immune cells that infiltrate gastrointestinal mucosa, as in inflammatory intestinal disease (IBD), secrete protumorigenic cytokines like TNF-*α*, IL-1, and IL-17 to increase NF-*κ*B activity and to increase colon cancer risk [76]. The recurrent upregulation of the NF-*κ*B signaling trail in carcinoma provides a stringent microenvironment that is essential either for tumor activation or for tumor growth, or collectively [77]. We noticed that the ruthenium-phloretin complex treatment module effectively decreased NF-*κ*B intensity in the colon carcinoma cells (Figure 5(e), A–G), hence denoting that ruthenium-phloretin treatment influences the inflammatory pathways in colon cancer. MMPs have emerged as essential regulatory proteins equally in pro- and anti-inflammatory pathways. MMP production and activation are usually enhanced in any tissue damage and inflammatory disease cycle, so there is considerable indication that these proteinases work primarily in inflammation to alter the infiltration of leukocytes, either by barrier function control or by cytokine/chemokine activity [78]. Our study provides conclusive evidence that ruthenium-phloretin treatment regulates MMP-9 expressions in inflammation-associated colon cancer (Figure 5 (vi) A-G).

Recent studies indicate that the two common hallmarks of tumors are altered redox balance and abrogate redox signaling which are sturdily associated with malignancy and resistance to treatment [79]. Thus, it can be expected that the upregulation of SOD, GSH, and CAT would result in the increment of the H_2_O_2_ level in the mitochondria, which is an important signaling molecule and a “reactive oxygen species” [80]. Several studies have directed that mitochondrial H_2_O_2_ is a direct and effective apoptotic process inducer [81]. Treatment of ruthenium-phloretin significantly elevated expressions of SOD, CAT, and GSH in colon cancer cells, probably by stimulating the ROS to instigate the apoptotic events (Figure 6(c)).

Cell growth is a necessity for the progression of cancer at primary and metastatic sites, and PCNA is a crucial factor for DNA replication; thus, the inhibition of PCNA is considered to be a viable anticancer strategy [82]. Our study reveals that the carcinogen control animals exhibited an upsurge in the amount of cells labeled with PCNA through declining AI (apoptotic index) (Table 9), indicating cellular proliferation and a greater number of ACF multiplicity within the colonic mucosa. On the other hand, a decrease of cells labeled with PCNA and consequent increment of AI were observed post treatment with the ruthenium-phloretin complex. In summary, the ruthenium-phloretin complex is accountable for the p53 intervene apoptosis in the colon carcinoma, instigated by instigation of the intrinsic apoptotic trail facilitated by the Bcl-2 and Bax and at the same time downregulating the Akt/mTOR with NF-*κ*B/MMP-9-regulated inflammatory pathways. Besides, the complex furthermore exhibits an antiangiogenic process by decrementing the VEGF biomarkers, allowing a decline of multiplicity of ACF in the colon mucosa. Moreover, the complex fruitfully demonstrated the intense activity on the free radical scavenging capabilities of colon carcinoma cells owing to the commencement of mitochondria-related reactive oxygen species by the regulation of p53. The diminution of PCNA due to the stimulation of p53 further heightens apoptosis by minimizing cellular proliferation.

The present alternative approach to colon cancer therapy shows higher efficiency at significantly lesser doses which offers insignificant toxicity and no additional adverse effects, as documented by our study. The outcomes give considerable proof that low dosages of ruthenium-phloretin chemotherapy could interrupt, revoke, or suspend the succession of colon carcinoma by finding the biomarkers corelated with the initiation of the apoptotic process in colon carcinoma and altering intrinsic apoptosis along with the antiangiogenic pathway, hence fulfilling the role of a prospective candidate in cancer chemotherapeutics in the vicinity of the future.

## Figures and Tables

**Table 1:** FTIR spectrum of phloretin and ruthenium-phloretin complex (band position cm^−1^).

Compound	v(O-H)	v(C=C)	v(C-O)	v(C-OH)	v(M-O)
Phloretin	3221.01		1248.92	980.13	
Ruthenium-phloretin	3215.23	1571.75	1240.53	972.67	614.22

**Table 2:** Chemical shift (*δ* (ppm)) of free phloretin and ruthenium-phloretin complex.

Compound	3-OH	5-OH	7-OH	2H-*β*	2H-*α*	9-OH	5-H	12-H	17-OH	15-H
Phloretin	9.18	12.30	10.56	2.86	3.20	3.41	5.80	3.26	7.02	6.69
Ruthenium-phloretin		12.26	10.40	2.83	3.21		5.81	3.23	7.01	6.67

**Table 3:** Hematological finding of male Balb/c mice for oral subacute toxicological analysis.

Parameters (±SEM)	Control	Ru-phl (300 mg)	Ru-phl (100 mg)	Ru-phl (50 mg)	Ru-phl (25 mg)
Hemoglobin (%)	12.22 ± 0.013	13.16 ± 0.01	12.45 ± 0.02	12.04 ± 0.03	12.28 ± 0.0410
Total RBC(10^6^/*μ*)	4.26 ± 0.001	5.01 ± 0.002^#^	4.53 ± 0.001	4.31 ± 0.002	4.60 ± 0.001
Platelet count (10^5^/*μ*)	2.95 ± 0.001	4.25 ± 0.03	2.65 ± 0.004	2.58 ± 0.001	2.78 ± 0.002
WBC (10^3^/*μ*)	8.64 ± 0.02	13.11 ± 0.02^#^	11.53 ± 0.02	6.3 ± 0.04	3.26 ± 0.01
MCV (fl)	87.96 ± 0.03	90.37 ± 0.02	84.30 ± 0.01	88.91 ± 0.03	87.14 ± 0.02
MCH (pg)	27.48 ± 0.02	29.36 ± 0.05	27.03 ± 0.02	28.72 ± 0.01	28.07 ± 0.02
MCHC (%)	32.58 ± 0.01	32.83 ± 0.01	31.03 ± 0.02	31.42 ± 0.01	31.76 ± 0.02
Neutrophil (%)	27.88 ± 0.20	30.58 ± 0.01	24.37 ± 0.48	27.29 ± 0.33	26.79 ± 0.01
Eosinophil (%)	2.15 ± 0.01	6.87 ± 0.01	6.43 ± 0.01	5.58 ± 0.06	1.42 ± 0.02
Monocyte (%)	1.44 ± 0.05	1.58 ± 0.01	1.28 ± 0.02	1.86 ± 0.07	1.20 ± 0.03
Basophil (%)	0.0 ± 0.0	0.0 ± 0.0	0.0 ± 0.0	0.0 ± 0.0	0.0 ± 0.0

Standard error of the mean is calculated as the ratio of standard deviation (SD) to the square root of entire subjects. Results were analyzed by *t*-test and ANOVA (one-way), and it was established by Dunnett's multiple comparison test. MCV: mean corpuscular volume; MCH: mean corpuscular hemoglobin; MCHC: mean corpuscular hemoglobin concentration; RBC: red blood cell; WBC: white blood cell. ^#^Level of significance at *p* < 0.05, as compared to the control group.

**Table 4:** Hematological finding of female Balb/c mice for oral subacute toxicological analysis.

Parameters (±SEM)	Control	Ru-phl (300 mg)	Ru-phl (100 mg)	Ru-phl (50 mg)	Ru-phl (25 mg)
Hemoglobin (%)	12.14 ± 0.022	13.16 ± 0.12	12.08 ± 0.04	12.34 ± 0.024	13.02 ± 0.15
Total RBC (10^6^/*μ*)	4.18 ± 0.05	5.06 ± 0.04^#^	4.50 ± 0.002	4.32 ± 0.004	4.60 ± 0.003
Platelet count (10^5^/*μ*)	2.95 ± 0.002	3.21 ± 0.005	2.63 ± 0.002	2.68 ± 0.002	2.88 ± 0.002
WBC (10/*μ*)	8.62 ± 0.05	13.16 ± 0.02^#^	11.84 ± 0.02	5.37 ± 0.03	3.84 ± 0.02
MCV (fl)	87.68 ± 0.02	90.28 ± 0.01	89.34 ± 0.05	88.96 ± 0.03	87.12 ± 0.23
MCH (pg)	27.21 ± 0.17	30.08 ± 0.03	29.38 ± 0.03	28.69 ± 0.01	27.14 ± 0.26
MCHC (%)	30.20 ± 0.044	31.64 ± 0.05	32.68 ± 0.05	30.26 ± 0.02	30.62 ± 0.03
Neutrophil (%)	28.60 ± 0.14	32.24 ± 0.05	24.80 ± 0.20	26.80 ± 0.20	24.00 ± 0.31
Eosinophil (%)	2.14 ± 0.09	1.20 ± 0.12	6.08 ± 0.04	5.08 ± 0.04	1.02 ± 0.02
Monocyte (%)	1.02 ± 0.02	2.32 ± 0.09	1.00 ± 0.04	1.05 ± 0.06	2.00 ± 0.01
Basophil (%)	0.0 ± 0.0	0.0 ± 0.0	0.0 ± 0.0	0.0 ± 0.0	0.0 ± 0.0

Standard error of the mean is calculated as the ratio of standard deviation (SD) to the square root of entire subjects. Results were analyzed by *t*-test and ANOVA (one-way), and it was established by Dunnett's multiple comparison test. MCV: mean corpuscular volume; MCH: mean corpuscular hemoglobin; MCHC: mean corpuscular hemoglobin concentration; RBC: red blood cell; WBC: white blood cell. ^#^Level of significance at *p* < 0.05, as compared to the control group.

**Table 5:** Serum biochemical finding in male Balb/c mice for 28 days of repeated-dose oral subacute toxicity study.

Parameters (±SEM)	Control	Ru-phl (300 mg)	Ru-phl (100 mg)	Ru-phl (50 mg)	Ru-phl (25 mg)
Aspartate aminotransferase (AST)	39.09 ± 0.11	50.11 ± 0.04^#^	40.02 ± 0.03	35.20 ± 0.05	30.05 ± 0.02
Alanine aminotransferase (ALT)	31.23 ± 0.03	50.01 ± 0.01^#^	45.03 ± 0.02	37.15 ± 0.06	34.04 ± 0.05
Alkaline phosphatase (ASP)	352.7 ± 0.12	495.3 ± 0.06^#^	365.2 ± 0.25	353.4 ± 0.16	221.4 ± 0.06
Blood urea nitrogen (mg/dl)	18.01 ± 0.02	30.20 ± 0.04^#^	28.24 ± 0.09	18.35 ± 0.01	17.08 ± 0.02
Creatinine (mg/dl)	0.61 ± 0.02	0.61 ± 0.001	0.61 ± 0.003	0.56 ± 0.01	0.57 ± 0.001
Glucose (mg/dl)	115.7 ± 0.17	127.9 ± 0.11^#^	115.3 ± 0.23	110.5 ± 0.13	105.4 ± 0.05
Cholesterol (mg/dl)	47.02 ± 0.04	50.04 ± 0.02	47.13 ± 0.09	43.12 ± 0.03	41.23 ± 0.02

Standard error of the mean is calculated as the ratio of standard deviation (SD) to the square root of entire subjects. Results were analyzed by *t*-test and ANOVA (one-way), and it was established by Dunnett's multiple comparison test. MCV: mean corpuscular volume; MCH: mean corpuscular hemoglobin; MCHC: mean corpuscular hemoglobin concentration; RBC: red blood cell; WBC: white blood cell. ^#^Level of significance at *p* < 0.05, as compared to the control group.

**Table 6:** Serum biochemical finding in female Balb/c mice for 28 days of repeated-dose oral subacute toxicity study.

Parameter (±SEM)	Control	Ru-phl (300 mg)	Ru-phl (100 mg)	Ru-phl (50 mg)	Ru-phl (25 mg)
Aspartate aminotransferase (AST)	31.24 ± 0.14	49.01 ± 0.01^#^	38.04 ± 0.05	33.13 ± 0.09	30.07 ± 0.03
Alanine aminotransferase (ALT)	31.23 ± 0.09	49.23 ± 0.02^#^	43.57 ± 0.03	37.04 ± 0.06	32.06 ± 0.08
Alkaline phosphatase (ASP)	352.4 ± 0.12	400.1 ± 0.34^#^	383.5 ± 0.23	310.7 ± 0.15	231.3 ± 0.09
Blood urea nitrogen (mg/dl)	17.08 ± 0.05	30.21 ± 0.02^#^	27.78 ± 0.02	26.53 ± 0.04	20.07 ± 0.03
Creatinine (mg/dl)	0.60 ± 0.004	0.60 ± 0.004	0.60 ± 0.002	0.54 ± 0.003	0.50 ± 0.002
Glucose (mg/dl)	114.6 ± 0.28	126.1 ± 0.24^#^	119.5 ± 0.34	119.4 ± 0.30	126.1 ± 0.20
Cholesterol (mg/dl)	46.07 ± 0.04	50.18 ± 0.05	46.03 ± 0.01	44.09 ± 0.06	42.03 ± 0.07

Standard error of the mean is calculated as the ratio of standard deviation (SD) to the square root of entire subjects. Results were analyzed by *t*-test and ANOVA (one-way), and it was established by Dunnett's multiple comparison test. MCV: mean corpuscular volume; MCH: mean corpuscular hemoglobin; MCHC: mean corpuscular hemoglobin concentration; RBC: red blood cell; WBC: white blood cell. ^#^Level of significance at *p* < 0.05, as compared to the control group.

**Table 7:** Measurement of ACF.

Groups	Amount of ACF/observation					Total amounts of ACF
	Observation 1	Observation 2	Observation 3	Observation 4	Observation 5	
Control	0.0 ± 0.0	0.0 ± 0.0	0.0 ± 0.0	0.0 ± 0.0	0.0 ± 0.0	0.0 ± 0.0
DMH+DSS	25 ± 1.22	37 ± 2.92	43 ± 3.21	32 ± 1.24	40 ± 4.32	177 ± 12.91
DMH+DSS+Ru-phl 50 mg/kg	10 ± 0.8	14 ± 3.1	11 ± 2.9	12 ± 3.3	14 ± 0.9	61 ± 11.0^∗∗^
DMH+DSS+Ru-phl 100 mg/kg	9 ± 0.7	12 ± 1.3	11 ± 2.2	10 ± 2.5	14 ± 0.5	56 ± 7.2^∗∗^
DMH+DSS+Ru-phl 200 mg/kg	4 ± 0.5	9 ± 1.2	10 ± 4.1	8 ± 0.9	9 ± 1.7	40 ± 8.4^∗∗∗^
DMH+DSS+100 mg/kg ruthenium	20 ± 2.5	25 ± 2.04	28 ± 1.9	31 ± 3.7	20 ± 1.7	124 ± 11.84^∗^
DMH+DSS+100 mg/kg phloretin	21 ± 1.9	27 ± 3.8	18 ± 0.7	19 ± 1.5	14 ± 0.6	99 ± 8.5^∗^

Values are expressed as the mean ± S.E.M., *n* = 6. Results were analyzed by *t*-test and ANOVA (one-way), and it was established by Dunnett's multiple comparison test. ^∗^*p* < 0.05, level of significance was observed between ruthenium 100 mg/kg, phl 100 mg/kg, and DMH treated with DSS group; ^∗∗^*p* < 0.05, level of significance was observed between Ru-phl 50 mg/kg and Ru-phl 100 mg/kg, and DMH treated with DSS group; and ^∗∗∗^*p* < 0.01, level of significance was observed between Ru-phl 200 mg/kg and DMH treated with DSS group. Five separate observations denote the presence of ACF from five different animals of each group. Approximately, 100 cells were counted per field, and 3 fields were examined per slide, and 3 slides were examined per group.

**Table 8:** Immunohistochemical analysis of Bax, Bcl-2, NF-*κ*Β, and MMP-9 in colon carcinoma.

Groups	Bax^§^	Bcl-2^§^	NF-*κ*Β^§^	MMP-9^§^
Control	11.2 ± 0.8	6.9 ± 0.2	8.2 ± 0.6	9.1 ± 0.4
DMH+DSS	4.5 ± 0.8	25.4 ± 1.8	21.9 ± 0.5	23.4 ± 0.7
Ru-phl 50 mg/kg	5.3 ± 0.2^∗∗^	18.4 ± 0.1^∗∗^	17.5 ± 0.2	20.6 ± 0.6
Ru-phl 100 mg/kg	8.1 ± 0.3^∗^	11.6 ± 0.2^∗∗^	10.3 ± 0.4^∗∗^	14.2 ± 0.5^∗∗^
Ru-phl 200 mg/kg	11.7 ± 1.8^∗^	7.3 ± 0.5^∗^	8.4 ± 0.6^∗^	9.5 ± 0.1^∗^
Ruthenium 100 mg/kg	5.9 ± 0.1	17.7 ± 0.8^∗∗^	15.8 ± 0.3^∗∗^	19.5 ± 0.7^∗∗^
Phloretin 100 mg/kg	9.2 ± 0.8^∗∗^	12.7 ± 0.2^∗^	13.8 ± 0.6^∗^	12.1 ± 0.9^∗^

^§^Score designated by the results of 6 slides per animal and 6 animals per group, mean ± S.E. (*n* = 6). Each area was chosen randomly for assessment of the percentage of immune-positive cells. ^∗^Level of significance was observed between treated and DMH treated with DSS group (*p* < 0.01). ^∗∗^Level of significance was observed between treated and DMH treated with DSS group (*p* < 0.05).

**Table 9:** Apoptosis and cell proliferation in colon.

Groups	PCNA-LI^§^	AI (%)^§^	*R* = PCNA‐LI/AI
Normal control	22.08 ± 0.4	0.17 ± 0.02	134.12 ± 0.2
DMH+DSS	37.5 ± 1.3	0.08 ± 0.03	468.75 ± 0.5
Ru-phl 50 mg/kg	27.7 ± 0.4	0.07 ± 0.02	395.71 ± 0.1
Ru-phl 100 mg/kg	21.2 ± 0.8^∗∗^	0.12 ± 0.04^##^	176.66 ± 0.2^$$^
Ru-phl 200 mg/kg	17.5 ± 0.2^∗^	0.13 ± 0.05^#^	134.61 ± 0.9^$^
Phloretin 100 mg/kg	20.9 ± 0.15^∗∗^	0.10 ± 0.03^##^	209.00 ± 0.7^$$^
Ruthenium 100 mg/kg	25.8 ± 0.12	0.11 ± 0.08	234.54 ± 0.7

LI = labelling index; PCNA-LI is represented as the percentage of PCNA-labelled cells to the ratio total number of cells counted; AI = apoptotic index. *R* is represented as PCNA-LI of to the ratio AI. AI was calculated as the percentage of TUNEL-positive cells/total number of cells counted. Values represent the mean ± S.E.^§^The total number of six slides was evaluated per rat. Each field consisted of approximately 500 cells. ^∗^Level of significance between PCNA-LI of Ru-phl 200 mg/kg vs. DMH treated with DSS animals (*p* < 0.01). ^∗∗^Level of significance between PCNA-LI of Ru 100 mg/kg, Ru-phl 100 mg/kg vs. DMH treated with DSS animals (*p* < 0.05). ^#^Level of significance between AI of Ru-phl 200 mg/kg vs. DMH treated with DSS animals (*p* < 0.01). ^##^Level of significance between AI of Ru 100 mg/kg, Ru-phl 100 mg/kg vs. DMH treated with DSS animals (*p* < 0.05). ^$^Level of significance between *R* of Ru-phl 200 mg/kg vs. DMH treated with DSS animals (*p* < 0.01). ^$$^Level of significance between *R* of Ru 100 mg/kg, Ru-phl 100 mg/kg vs. DMH treated with DSS animals (*p* < 0.05).

## Data Availability

The data used to support the findings of this study are available from the corresponding author upon request.

## Abbreviation

DMSO: Dimethyl sulfoxide
TPTZ: 2,4,6-Tri(2-pyridyl)-striazine
FT-IR: Fourier transform infrared spectroscopic analysis
XRD: X-ray diffraction spectroscopy
VEGF: Vascular endothelial growth factor
FRAP: Ferric reducing antioxidant power
DPPH: 1,1-Diphenyl-2-picrylhydrazyl
ABTS: 2,2-Azino-bis(3-ethylbenzothiazoline-6-sulfonate)
NMR: Nuclear magnetic resonance
MTT: 3-(4,5-Dimethyl thiazole-2-yl)-2,5-diphenyltetrazolium bromide
PCNA: Proliferating cell nuclear antigen
mTOR: Mammalian target of rapamycin
ACF: Aberrant crypt foci
IR: Infrared spectroscopy.

## Supplementary Materials

Supplementary Materials (1) Page 1: certificate from the Institutional Ethical Committee denoting the study number and the number of animals used. (2) Page 2: full blot of protein pro-caspase-3 in HT-29 cells after treatment with the ruthenium-phloretin complex. (3) Page 2: full blot of protein active caspase-3 in HT-29 cells after treatment with the ruthenium-phloretin complex
