# Supplementary material for: Decrypting the Molecular Mechanistic Pathways Delineating the Chemotherapeutic Potential of Ruthenium-Phloretin Complex in Colon Carcinoma Correlated with the Oxidative Status and Increased Apoptotic Events
Source: Oxid Med Cell Longev. 2020 May 31;2020:7690845. doi: 10.1155/2020/7690845 (PMC7281810; doi:10.1155/2020/7690845)

NSHM College of Pharmaceutical Technology
Kolkata – 700 053.

INSTITUTIONAL ANIMAL ETHICS COMMITEE

Regd. No: 1458/PO/a/11/CPCSEA

hairperson
r. Subhasis Maity

PCSEA Nominee
r. A. Sikdar

embers
r. Tapan k. Barman
r. Musfiqua Mookerjee
r. Tapan K. Chatterjee
r. Supriya Mana

cial activist
r. Kunal Bhattacharjee

eterinarian
r. Sourav Banerjee

Ref.: HCG/Pharmacol/2019/05 Date: 10/04/2019

This is to certify that the thesis entitled "The investigation of Ruthenium-Phloretin complex in colon carcinoma:a molecular approach" and related experimental work will be carried out through the animals subjected to the guidelines set by animal welfare division, Ministry of Environment, Govt. of India throughout this study. 60 mice and 35 rats have been approved for experimental purposes. The guidelines for the care and the use of laboratory animals were strictly followed during the experiment and approved by the Institutional Animal Ethical Committee (IAEC).

Chairperson

Institutional Animal Ethics Committee
NSHM College of Pharmaceutical Technology

Pro Caspase-3 Full Western Blot

Con 36.56μm 73.13 μm 109.69 μm

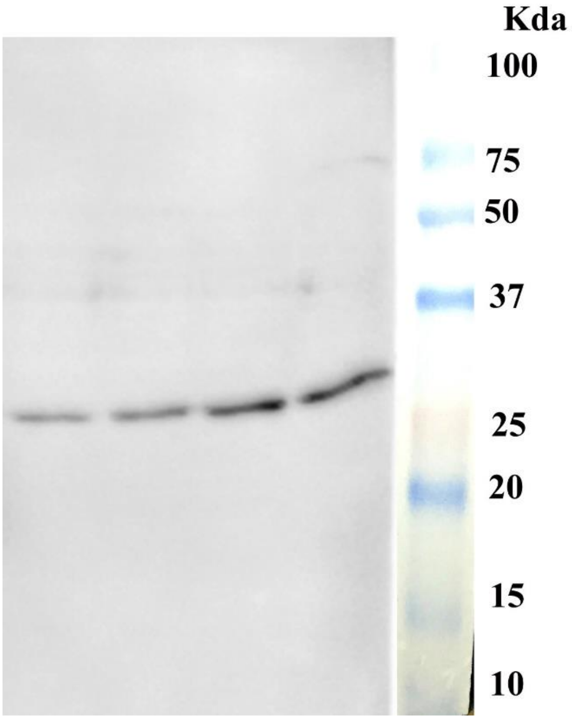

Active Caspase-3 Full Western Blot

Con 36.56µm 73.13 µm 109.69 µm

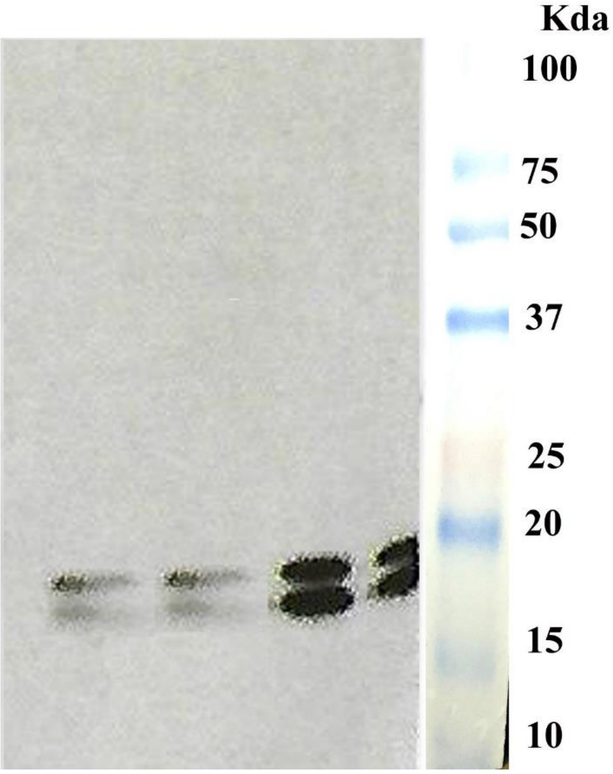

Supplement: Supplementary Materials — (1) Page 1: certificate from the Institutional Ethical Committee denoting the study number and the number of animals used. (2) Page 2: full blot of protein pro-caspase-3 in HT-29 cells after treatment with the ruthenium-phloretin complex. (3) Page 2: full blot of protein active caspase-3 in HT-29 cells after treatment with the ruthenium-phloretin complex [file 7690845.f1.pdf]
